# Assessing Pollution with Heavy Metals and Its Impact on Population Health

**DOI:** 10.3390/toxics13010052

**Published:** 2025-01-12

**Authors:** Youssef Saliba, Alina Bărbulescu

**Affiliations:** 1 Doctoral School, Technical University of Civil Engineering of Bucharest, 122-124 Bd. Lacul Tei, 020396 Bucharest, Romania; youssefsaliba@gmail.com; 2 Department of Civil Engineering, Transilvania University of Brașov, 5 Turnului Str., 500152 Brașov, Romania

**Keywords:** multivariate analysis, pollution indicator, PCA, t-SNE, FA, *HI*

## Abstract

Pollution is one of the most important issues currently affecting the global population and environment. Therefore, determining the zones where stringent measures should be taken is necessary. In this study, Principal Component Analysis (PCA), Factor Analysis (FA), and t-distributed Stochastic Neighbor Embedding (t-SNE) were utilized for dimensionality reduction and clustering of data series containing the concentration of 10 heavy metals collected at 14 locations. The Hazard Quotient (*HQ*) and Hazard Index (*HI*) were utilized to determine the non-carcinogenic risk to the population in the studied zones. The highest concentrations of metals in the samples were those of Fe, Zn, Mn, and Cr. PCA indicated that Fe and Zn (Co and Cd) had the highest contribution on the first (second) Principal Component (PC). FA showed that the three-factor model is adequate for explaining the variability of pollutant concentrations. The factor loadings revealed the strength of association between variables and factors, e.g., 0.97 for Zn, 0.83 for Cr, and 0.99 for Co. *HQ* for ingestion, ${HQ}_{ing},$ was the highest for Fe (between 6.10 × 10^−5^ and 2.57 × 10^−4^). *HQ* for inhalation, ${HQ}_{inh}$, was the biggest for Mn (from 1.41 × 10^−3^ to 1.95 × 10^−3^). *HI* varied in the interval [0.172, 0.573], indicating the absence of a non-carcinogenic risk. However, since values above 0.5 were determined at four sites, continuous monitoring of the pollution in the sampling locations is necessary.

## 1. Introduction

Heavy metals are widely recognized as significant environmental contaminants [1]. The sources of heavy metals can be categorized as anthropogenic and natural. The first category, including mining, agriculture, vehicle emissions, smelting operations, fossil fuel combustion, and other industrial activities, mainly contributes to heavy metal pollution, especially in urban zones [2,3,4,5]. The utilization of chemical fertilizers and pesticides, coal burning, and metal extraction releases heavy metals like arsenic (As), copper (Co), cadmium (Cd), lead (Pb), and mercury (Hg) into the air, soil, and water. Chromium (Cr), nickel (Ni), cobalt (Co), zinc (Zn), and copper (Cu) released into the atmosphere can also pose severe risks to human and environmental health, even at trace levels [6]. While some metals are essential for specific biological functions in trace amounts, excessive accumulation can seriously harm human health [7]. Their toxic effects, ranging from chronic diseases to neurological damage, are intensified by their ability to accumulate in the environment and living organisms over time [8,9,10,11,12].

The second category, including natural sources (forest fires, volcanic eruptions, sea salt sprays, rock weathering, and wind-borne soil particles), also contributes to heavy metal levels in the atmosphere. However, their impact is generally lower than that of the first category [13].

Recent studies [14,15,16] have indicated that urban areas are especially vulnerable to high levels of heavy metal contamination, vehicle emissions being a significant contributor to the pollution of cities, releasing a complex mixture of particles and gases [4,5,17,18]. Road dust—a mix of particles from soil, vehicle emissions, and atmospheric deposition—is one of the most common and dangerous paths for heavy metal exposure in urban settings. It often contains metals from tire wear, brake pads, exhaust fumes, and engine components. The wear and tear on vehicles’ mechanical parts, tires, and industrial processes significantly contribute to road dust pollution, introducing metals such as lead, nickel, zinc, and copper [17,18,19,20,21].

The accumulation of road dust containing heavy metals affects inhaled air quality and poses risks to people living in such contaminated environments, as well as ecological equilibrium [22,23].

The dust from the desert regions (Sahara in Africa, the Middle East and Arabian Peninsula, and Central Asian Deserts) constitutes another significant atmospheric pollution source. Dust is transported long distances by strong winds and can impact air quality and climate far from its origin [24,25,26]. Being located in a desert region with vast areas of sand dunes (particularly in the Rub’ al Khali), the United Arab Emirates (UAE) is one of the countries that experiences dust storms due to the winds (Shamal) that blow from the northwest, especially during the spring and summer [27,28]. Rapid urbanization and infrastructure building also generate large amounts of dust.

Modeling real-life phenomena necessitates advanced mathematical methods. In the context of increasing pollution from various sources, scientific inquiry into the impact of pollution is an iterative process involving hypothesis formulation, data collection, and analysis. The complexity of this phenomenon necessitates performing multivariate analysis, which is indispensable for extracting meaningful information from the collected datasets. Examining the relationships between variables provides a comprehensive understanding that univariate methods often fail to achieve. Still, while robust, multivariate analysis presents cognitive and computational challenges [29,30].

Machine learning (ML) methods, like XGBoost [31], Random Forest (RF) [31,32,33], ANFIS and ANN [34], Support Vector Regression [32], and Regression Trees [35], have been extensively used to model soil contamination. Unsupervised classification techniques, including K-means [36], DBSCAN [37], Hierarchical Clustering [38], Mean Shift [39], Support Vector Machine [40], Naive Bayes Classifier [41], OPTICS [42], etc., have proven their efficiency in various classification problems. Previous studies have indicated that combining multivariate statistics and unsupervised classification techniques can deepen the understanding of pollution sources and their spatial distribution [16,26,43,44].

In the above context, this article emphasizes algebraic concepts, minimizing advanced mathematics while maintaining a quantitative approach, which is then applied to (1) analyzing the heavy metal concentrations in dust collected in the Dubai area and (2) determining the non-carcinogenic health risk to the population living in the neighborhoods of the sampling sites. For the first goal, dimensionality reduction, clustering, and investigating the correlations between the concentrations of the metals in dust samples were performed. The second goal was achieved by computing *HQ* indices.

By maintaining a critical perspective and effectively integrating multivariate methods with the *HQ* analysis, one can uncover meaningful insights into metal pollution in the region, find the locations where advanced de-pollution methods should be used, and provide a background for making decisions to preserve a clean environment.

## 2. Data Series and Methodology

### 2.1. Study Region and Data Series

The United Arab Emirates (UAE) belongs to a dry and warm subtropical climate. An extended surface of the country is covered by sand dunes. From May to October, daytime temperatures vary between 35 and 50 °C, while they are generally between 20 and 35 °C at midday in the rest of the year. In the desert interior, summer (winter) ground temperatures can reach 70 °C (0 °C or below). The annual precipitation is under 100 mm. Rainfall is irregular, mostly during winter, significantly varying by location and year. Coastal areas also experience dew and fog that provide additional moisture. Sandstorms are frequent, especially in the summer [45].

**Figure 1:**
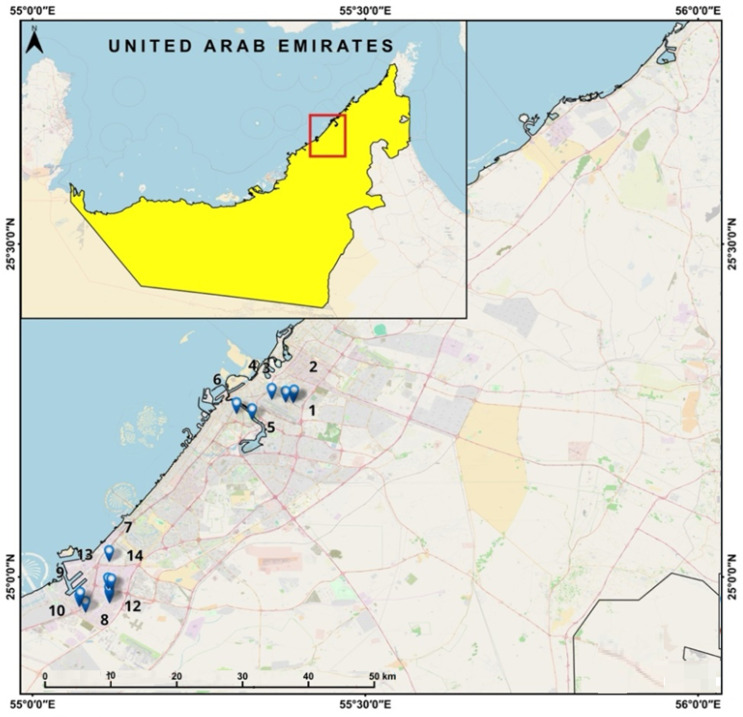
Study area and the sampling locations.

The study area is located in the Dubai Emirate (Figure 1). The soils are mainly calcareous and sandy and deficient in organic matter. Along the coast, in depressions and low-lying zones, the soils are characterized by high salinity, whereas in the inner desert, they are sodic or saline [45].

The dataset consists of the series of measured concentrations of heavy metals (Ba, Co, Cu, Cd, Cr, Fe, Mn, Cr, Pb, Zn) extracted from analyzing the dust samples collected in 14 locations in Dubai (Figure 1), the United Arab Emirates, following the procedure from [21].

### 2.2. Methodology

The data series was subject to statistical analysis to determine the series characteristics. The basic statistics included minimum (min), maximum (max), mean, standard deviation (stdev), and outliers’ detection.

Principal Component Analysis (PCA), Factor Analysis (FA), and t-distributed Stochastic Neighbor Embedding (t-SNE) were used to address the contamination extent in various locations and to group those sites based on their similarities. PCA and t-SNE are unsupervised algorithms. PCA is a deterministic method that linearly reduces the dimensions of the dataset. t-SNE is a non-linear, randomized algorithm that maps a high-dimensional dataset to a lower-dimensional space. Its output is typically employed for visualization. These techniques are explained in detail in Section 2.2.1 and Section 2.2.3

R.4.3.2 software was employed to carry out the study. It offers a comprehensive suite of packages and functions designed for multivariate analysis, **psych** for FA [46], **FactorMineR**, **factoextra**, and **MASS** [47,48,49] for PCA, and NbClust [50] for clustering. Its robust computational capabilities and visualization tools make R ideal for handling complex datasets. Additionally, we used maximum likelihood to fit various models.

This approach will enhance our understanding of the underlying methodologies and provide flexibility in diverse analytical scenarios. By leveraging R, we can efficiently implement and interpret multivariate techniques, ensuring accurate and insightful pollution analysis.

#### 2.2.1. Principal Component Analysis

PCA allows for straightforward analysis without the significant loss of information by reducing the dimensionality of a dataset while retaining its structure [51,52]. It transforms potentially correlated variables into uncorrelated orthogonal Principal Components (PCs), linear combinations of the original variables, ordered by the proportion of variance they explain. PCA can reveal hidden patterns and relationships among variables. It is performed on the correlation matrix, especially when the variables (the heavy metals’ concentrations, in our case) are measured on different scales. The standardization step makes the procedure robust against the influence of variables with more significant variances.

To perform PCA on a multivariate vector $x=\left(x_{1},x_{2},\ldots ,x_{p}\right)$, the eigenvalues and eigenvectors of the sample variance matrix S provide the estimations of the variances and directions of the PCs. The *j*th PC is given by:

(1) $$y_{j}=e_{j}^{\prime }x$$

where $e_{j}^{\prime }$ is the *j*-th eigenvector.

The *j*th PC captures a fraction of the total variance equal to

(2) $$t_{j}=\frac{\lambda_{j}}{\sum_{k=1}^{p}\lambda_{k}}$$

where $\lambda_{j}$ is the *j*th eigenvalue.

If a few components capture a significant percentage of the total variance, the data’s dimensionality can be reduced with minimal loss of information.

Different approaches can be utilized to select the optimum number of PCs [53,54]. An eigenvalue greater than 1 (often serving as a threshold for selecting a PC) suggests that the corresponding PC accounts for more variance than any of the original standardized variables. A PC whose eigenvalue $\lambda_{j}$ is greater than 1 (the average eigenvalue when working with standardized variables) is kept, while the others are discarded.

An alternative procedure uses a Scree Plot that displays the eigenvalues from the highest to the lowest. It allows the selection of the PCs until the corresponding eigenvalues start to level off [51,55]. Another approach is to retain the number of components that collectively explain a predetermined fraction of the variance (usually 70% or 80%). However, there is no universally accepted method for deciding the optimal number of PCs to retain.

Information Criteria like Akaike (*AIC*) and Bayesian (*BIC*) [56,57] can also be utilized to select the best number of PCs. If *n* and *k* are the sample size and number of components, respectively, $L_{k}$ is the log likelihood for PCs, and ${AIC}_{k}$ and ${BIC}_{k}$ are defined by (3) and (4), respectively:

(3) $${AIC}_{k}=-2ln{(L}_{k})+2k,$$

(4) $${BIC}_{k}=-2ln{(L}_{k})+kln(n).$$

The optimal number of PCs is the one for which ${AIC}_{k}$ or ${BIC}_{k}$ is minimized, balancing model fit and complexity. Based on *AIC*, a new PC can be added when its corresponding eigenvalue, $\lambda_{k+1},$ satisfies the inequality $\lambda_{k+1}>exp(-2/n).$ Considering *BIC*, a new PC is added when $\lambda_{k+1}>n^{1/n}$.

#### 2.2.2. Factor Analysis

FA is a technique used to discover groups of variables, called factors, which seem to act together. The FA model expresses each observed variable as a linear combination of the underlying factors plus an error. Estimating the factors and loadings relies on the hypothesis that the factors and errors means are zero, the factors and errors are uncorrelated, and the factor vector has a variance equal to 1. In these hypotheses, the covariance matrix can be decomposed into a part that captures the common variance explained by the factors and another representing the unique variance unexplained by each.

We fit an FA model to uncover the latent factors that influence the concentrations of various elements in the dust samples and understand any spatial patterns or regional influences on these concentrations. The factor loadings obtained from the model will show how the concentration of each element relates to these latent factors, helping us identify groups of elements that are influenced by the same underlying process.

Various methods can be employed to check if the chosen number of factors adequately explains the variability in the data, including the Parallel Analysis (PA) and Very Simple Structure (VSS) criteria [58]. The *BIC* and *AIC* criteria can be utilized to find the number of factors (*m*) that balance the model fit and complexity [59].

In the FA, by comparing the eigenvalues of the observed data $\lambda_{j}$ with those obtained from randomly generated data ${\bar{\lambda }}_{j}$ and selecting the factors that satisfy the relation $\lambda_{j}>{\bar{\lambda }}_{j}$, one can determine which factors explain more variance than would be expected by chance. In VSS, the number of factors (*m*) that maximize the VSS index is considered optimal [58].

*AIC* and *BIC* minimization was also utilized to determine the optimal number of factors. The formula used here, implemented in psych::fa()function in R, is slightly different than those in (3) and (4). *BIC* is defined by:

(5) $$BIC=\chi^{2}-2df$$

where $\chi^{2}$ is the chi-square statistic and df is the degree of freedom of the model.

$\chi^{2}$ is derived from the likelihood function representing the goodness of fit and df measures the model’s complexity.

We can formulate *AIC* in the same context by:

(6) $$AIC=\chi^{2}+2df.$$

Including more parameters in (6) will increase the *AIC*, favoring simpler models with better fit.

#### 2.2.3. t-Distributed Stochastic Neighbor Embedding (t-SNE)

Another algorithm designed for dimensionality reduction, particularly useful for visualizing high-dimensional data by embedding it into two or three dimensions—t-SNE [60]—is based on probability distributions with random walk on neighborhood graphs to find the structure within the data. This technique aims to preserve the local structure of the data while also revealing global patterns.

t-SNE has been used in applications from various research domains, including genomics, medicine, bioinformatics, natural language processing, computer security, geology, and geochemistry [60,61,62,63,64,65,66].

The key concepts and mathematical formulation of t-SNE, providing an understanding of how the algorithm works and why specific methods are used, are presented in the following.

*The high-dimensional similarities* ensure the similarity between pairs of data points in the high-dimensional space. The similarity $p_{j|i}$ between vectors $x_{i}$ and $x_{j}$ is defined by:

(7) $$p_{j|i}=\frac{exp(-{\left||x_{i}-x_{j}|\right|}^{2}/2\sigma_{i}^{2})}{\sum_{k\neq i}exp(-{\left||x_{i}-x_{k}|\right|}^{2}/2\sigma_{i}^{2})}$$

where $\sigma_{i}$ is the bandwidth of the Gaussian centered at $x_{i}$.

This approach ensures that points closer in high-dimensional space have higher probabilities, reflecting their local similarity.

*Perplexity (Perp)* controls the effective number of nearest neighbors considered for each point [67]. Its values vary from 5 to 50 and should be less than the number of samples [68]. Perplexity balances the focus between local and global aspects of the data and is defined as

(8) $$Perp(P_{i})=2^{H(P_{i})},$$

where $H\left(P_{i}\right)$ is the Shannon entropy of the probability distribution ($P_{i}$) in bits:

(9) $$H(P_{i})=-\sum_{j}p_{j|i}{log}_{2}(p_{j|i}).$$

The *low-dimensional similarities* $q_{ij}$ are modeled using a t-distribution with one degree of freedom (or a Cauchy distribution) in the low-dimensional space.

*The crowding problem* [69] refers to the case when high-dimensional data points that are moderately far apart are placed too close in a lower-dimensional representation, leading to a loss of local structure. This situation occurs because, in lower-dimensional spaces, there is not enough “room” to maintain the correct relative distances for both close and moderately far points, leading to a loss of meaningful relationships.

*Kullback-Leibler Divergence (KL divergence)* [70] measures the difference between two probability distributions. In t-SNE, it quantifies the discrepancy between the high- and low-dimensional probability distributions *P* and *Q*. Minimizing *KL* divergence in t-SNE preserves local structure by ensuring that the high-dimensional and low-dimensional representations of the data have similar probability distributions. It means aligning *Q* closely with *P*, ensuring that points that are close together in *P* remain close in *Q*.

To find the optimal low-dimensional representation, we aim to minimize KL divergence using *gradient descent with momentum* (GD) [71] while eliminating local minima. The learning rate η is a hyperparameter that controls the size of the steps taken during GD. A smaller η makes the algorithm more stable but slower to converge, while a larger η speeds up convergence but risks overshooting the minimum.

The learning rate is often set to values such as 200 or 1000, but it can be adjusted based on the dataset and the specific requirements of the analysis. It controls the size of steps taken towards the loss function minimum.

To achieve optimal performance for t-SNE, it is essential to focus on the parameters that most significantly influence the algorithm’s performance, such as *Perp*, η, and α (the momentum term, usually between 0.5 and 0.9). Common practices suggest using standard values already mentioned in this section. Nevertheless, multiple methods, such as Bayesian optimization, cross-validation, and automated methods, can be applied. Here, we will present the Bayesian optimization method, in which we define three different objective functions that measure the quality of the resulting embedding.

Before analyzing the choice of the objective function, we will run the t-SNE algorithm using the element concentration data to get a general picture of the results. The number of lower dimensions will be 2, perplexity—3, maximum iterations—500, learning rate—50, and momentum—0.5. Finally, we will visualize the results.

*Quantitative analysis* assesses how well the optimization has separated the data points. The average distances within and between clusters were computed for this aim. KL divergence measures how one probability distribution diverges from a second expected probability distribution. By comparing the average intra-cluster distances (distances within the same cluster) and inter-cluster distances (distances between different clusters), we can determine if the optimized parameters lead to tighter, more distinct clusters. A significant reduction in intra-cluster distances and an increase in inter-cluster distances indicate that optimization effectively improves the separation and coherence of clusters.

*Element concentration analysis* enhances our understanding of the cluster profiles by examining the distribution of individual element concentrations within and between the clusters. By plotting the concentrations of each element for the identified clusters, we can discern patterns and differences in the dust composition across different geographical locations. This analysis is particularly useful for identifying which elements contribute most to the clustering observed in the t-SNE plot. It can reveal whether certain elements are prevalent in specific clusters, suggesting underlying environmental or geological factors influencing the dust composition.

### 2.3. Health Risk Assessment

The non-carcinogenic health risk for the population living in the research area was investigated by computing the average daily dose, *ADD* (mg/kg/day), of each metal, *k*, by ingestion (${ADD}_{k,ing}$), inhalation ${(ADD}_{k,inh})$, and dermal contact (${ADD}_{k,derm}$), using (10)–(12) [72,73,74,75].

(10) $${ADD}_{k,ing}=\frac{c_{k}\times R_{ing}\times EF\times ED}{BW\times AT}\times 10^{-6}$$

(11) $${ADD}_{k,inh}=\frac{c_{k}\times R_{inh}\times EF\times ED}{PEF\times BW\times AT},$$

(12) $${ADD}_{k,derm}=\frac{c_{k}\times SA\times SL\times ABS\times EF\times ED}{BW\times AT}\times 10^{-6}.$$

The notations are explained in Table 1, according to [76].

The reference dose for a metal *k* (${RfD}_{k}$) is the upper limit of the acceptable risk by daily exposure to that metal of the people (adults, in this study) during their entire life lifetime. When ${ADD}_{k,ing}$ < ${RfD}_{k,ing}$ (${ADD}_{k,inh}$ < ${RfD}_{k,inh}$ and ${ADD}_{k,derm}<{RfD}_{k,derm},$ respectively), no adverse effects are likely to appear. The values of the reference doses for each metal analyzed in this article are presented in Table 2. They are the most used in the scientific literature [73,74,75,76,77,78,79,80,81]. Extensive discussions on various *RfD* values are found in [77].

The Hazard Quotients for ingestion, inhalation, and dermal contact for a metal *k* (${HQ}_{k,ing},\;{HQ}_{k,ing},$ and ${HQ}_{k,derm})$ are computed by:

(13) $${HQ}_{k,ing}=\frac{{ADD}_{k,ing}}{{RfD}_{k,ing}},$$

(14) $${HQ}_{k,inh}=\frac{{ADD}_{k,inh}}{{RfD}_{k,inh}},$$

(15) $${HQ}_{k,derm}=\frac{{ADD}_{k,derm}}{{RfD}_{k,derm}}.$$

They express the non-carcinogenic risk by ingesting the metal *k,* its inhalation, or by dermal contact, respectively.

The Hazard Quotient with respect to the metal *k* is obtained by:

(16) $${{HQ}_{k}=HQ}_{k,ing}+{HQ}_{k,inh}+{HQ}_{k,derm}.$$

To evaluate the total non-carcinogenic risk for the exposed population, one may use the Hazard Index (*HI*) obtained by summing up the *HQ* by all paths for all *n* metals, so:

(17) $$\;\;\;\;\;\;\;\;\;\;HI=\sum_{k=1}^{n}{HQ}_{k}.$$

*HI* > 1 indicates a possible non-carcinogenic effect on human health.

## 3. Results and Discussion

### 3.1. PCA Results

**Figure 2:**
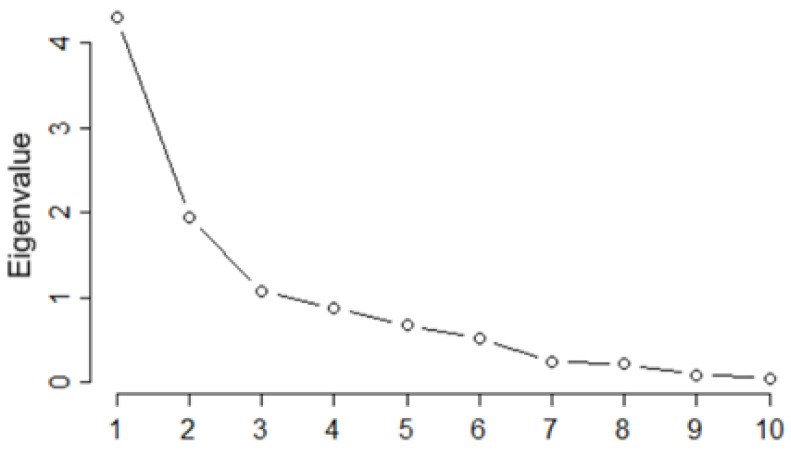
Scree plot.

The PCA was conducted for 14 individuals (locations) and 10 variables (the metals concentrations). The first four eigenvalues (ordered in decreasing order) had the values of 4.21, 1.94, 1.08, and 0.88 (Figure 2), explaining 43.08, 19.37, 10.80, and 8.81% of the variance, respectively. Therefore, the first three PCs accounted for 73.25% of the variability in the data, and the first four PCs explained 82.06% of the variability.

The first three PCs, with eigenvalues greater than 1, can be selected based on the Kaiser criterion. According to the cumulative variance explained (%), the first three or four PCs should be retained.

Table 3 presents the values of *AIC* and *BIC* corresponding to the first four components. Based on *AIC* and *BIC*, the optimal number of PCs to retain is four, which will be used in the following study.

**Figure 3:**
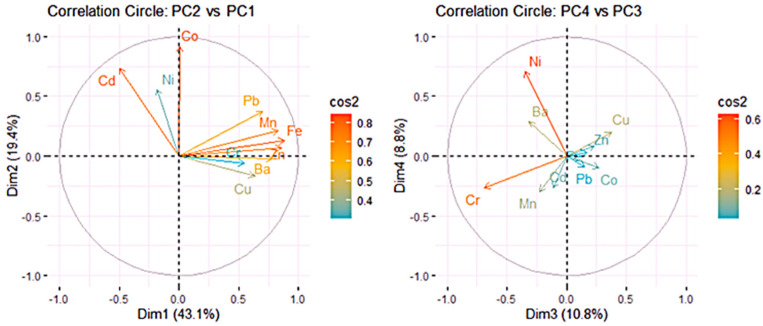
Correlation circles PC2 vs. PC1 (**left**) and PC4 vs. PC3 (**right**).

The relationships among all variables can be observed from the variable correlation circles (Figure 3). The positively correlated variables are clustered together, while those negatively correlated are located on opposite sides of the plot with respect to origin.

**Figure 4:**
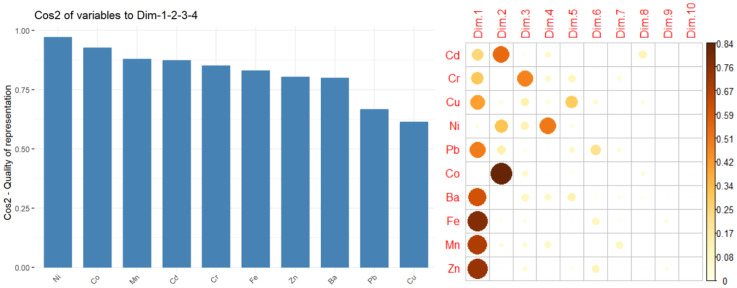
Quality of representation (**left**); contributions to Dim.1–Dim.10 (**right**).

The distance of variables from the origin indicates the quality of their representation on the factor map, with variables farther from the origin being better represented. In Figure 3 and Figure 4 (left), a high cos2 value signifies a good representation of the variable on the PC, placing the variable near the circumference of the correlation circle. Fe, Zn, and Mn are the best represented on PC1 and Co on PC2. Cr and Ni are best represented in the negative direction of PC3, and Ni is best represented on PC4.

Figure 4 (left) shows the quality of representation of the variables to the first four PCs. Ni, Co, Mn, and Cd, with cos2 values above 0.80 are the best represented. However, Pb and Cu, while still contributing to the explained variance, are worse represented (with cos2 > 0.625).

Variables that show strong correlations with the first four PCs (Dim.1 to Dim.4) play a significant role in explaining the variability within the dataset. The less significant variables could be removed to simplify the overall analysis. Figure 4 (right) indicates the following significant contributions to: Dim.1: Fe, Zn, Mn, Ba, Pb; Dim.2: Co, Cd, Ni; Dim.3: Cr, Cu, Ni; Dim.4: Ni. 

While the current analysis has focused on the contributions and significance of the variables (metals), it is equally important to understand how the individual observations (locations) are represented in the Principal Component space. PCA for individuals involves examining each sample’s scores (coordinates) on the PC. This analysis helps identify patterns, clusters, and outliers, providing insights into the relationships between sites based on the PC. By visualizing and interpreting the PCA results for individuals, we can gain a deeper understanding of the dataset’s structure from the perspective of the observation sites, complementing the variable-based analysis and offering a comprehensive view of the data.

**Figure 5:**
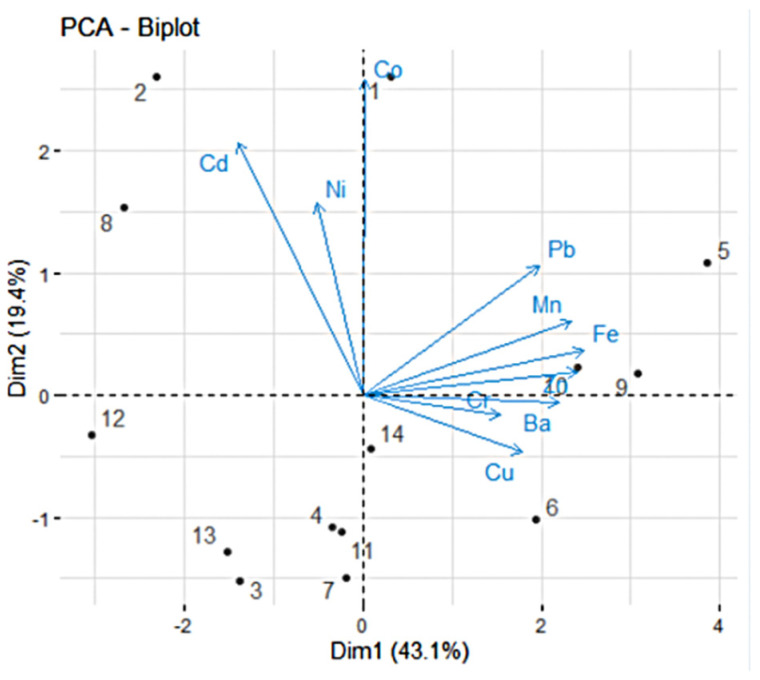
PCA biplot. The sites are represented by dots numbered from 1 to 14.

The biplot (Figure 5) summarizes both aspects—the contributions of the variables and individuals. Sites 5 and 9 are best represented on the positive side of PC1, while 12 is best represented on the opposing side. Sites 2 and 1 are best represented on the positive side of PC2, while 3, 7, and 14 are best represented on the negative part.

### 3.2. FA Results

We fit an FA model to determine the latent factors influencing the elements’ concentrations and to understand the spatial patterns or regional influences on these concentrations. The factor loadings show how each element concentration relates to the latent factors, helping us identify groups of elements influenced by the same underlying process. High loadings on a specific factor indicate that that factor significantly influences the element. First, we performed FA using the maximum-likelihood-as-fit method and fixed the number of factors to three. The results are presented in Table 4. Columns 2–4 contain the standardized loadings (ordered based on their decreasing importance) based on the correlation matrix. Columns 4–6 contain the communalities (h2), uniqueness (u2), and complexity (com), respectively.

The analysis shows that three factors (ML2, ML3, ML1) explain 64% of the total variance. Here is a quick breakdown of the results:

1. Factor loadings and variance:
  - Sum of squared (SS) loadings: ML2—2.22, ML3—1.77, ML1—1.74;
  - Proportion variance: ML2—0.29, ML3—0.18, ML1—0.17;
  - Cumulative variance: 64%;
  - Loadings: Indicate the strength of association between variables and factors, e.g., Zn (ML2: 0.97), Cr (ML3: 0.83), Co (ML1: 0.99);
  - h2 and u2: High communalities indicate variables well-explained by the factors. For example, Zn has h2 = 0.91 and u2 = 0.091, indicating that the factors explain 91% of its variance. The same is true for Mn.
  - Factor correlations: ML2-ML3: 0.54, ML2-ML1: 0.03, ML3-ML2: 0.54, ML3-ML1: −0.02.
2. Model fit indices:
  - Chi-square statistic: 11.23 (*p* < 0.88);
  - Root Mean Square of Residuals (RMSR): 0.07;
  - Tucker–Lewis Index (TLI): 4.006;
  - *BIC*: −35.27.
3. Factor score adequacy indicates a high reliability of factor scores:
  - Correlation of regression scores with factors: ML2 (0.97), ML3 (0.95), ML1 (1.00);
  - Multiple R-square of scores with factors: ML3 (0.95), ML1 (0.90), ML2 (0.99);
  - Minimum correlation of possible factor scores: ML3 (0.90), ML1 (0.80), ML2 (0.99).

**Figure 6:**
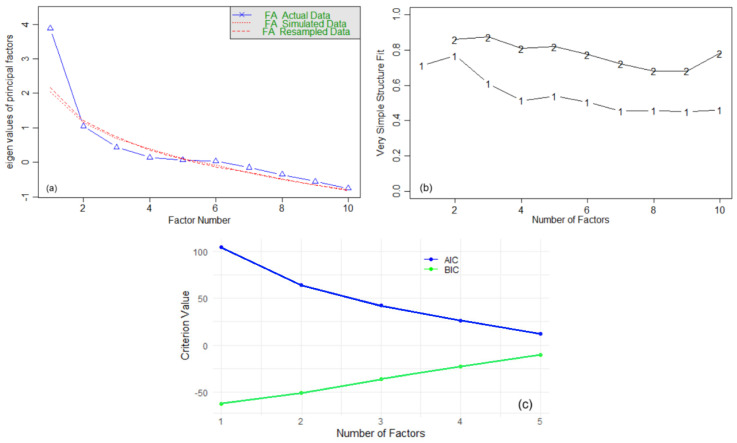
(**a**) Scree plot; (**b**) VSS; (**c**) *AIC* and *BIC* for FA.

These results indicate that the three-factor model is adequate for explaining the variability of pollutants’ concentrations, with high factor score reliability and good fit indices. However, the choice of *m* = 3 was arbitrary. Based on the Scree plot (Figure 6a), we should retain two factors.

Running the VSS (Figure 6b), we obtained the following output: Complexity 1 achieves a maximum fit of 0.77 with two factors, while Complexity 2 achieves a maximum fit of 0.87 with three factors. The minimum *BIC* = −63 was achieved with one factor, followed by the value of −51, with two factors. The results show that two factors balance simplicity and explanatory power well. Finally, we used the *AIC* and *BIC* criteria to determine the optimal number of factors. From Figure 6c, we observe that the *AIC* decreases consistently as the number of factors increases from one to five. It suggests that adding more factors improves the model fit, according to *AIC*. However, the *BIC* shows an increasing trend. *BIC* penalizes model complexity more heavily than *AIC*. Therefore, considering the balance between the model fit and its complexity, the optimal number of factors is two.

### 3.3. T-SNE Results

**Figure 7:**
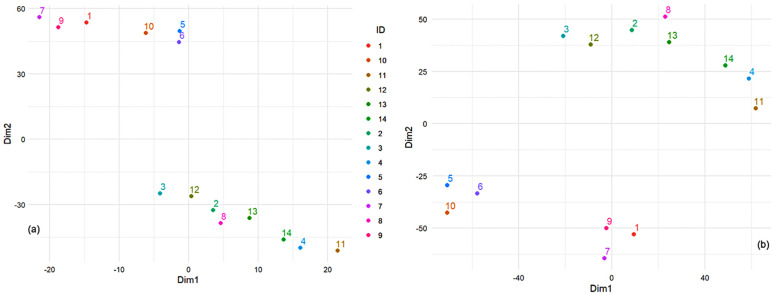
Results of t-SNE (**a**) before and (**b**) after optimization by the first criterion.

Before delving into the choice of the objective function, we ran the t-SNE algorithm using the element concentration data to get a general picture of the results. We chose the number of lower dimensions to 2, perplexity to 3, maximum iterations to 100, learning rate to 50, and momentum to 0.5. The t-SNE results (Figure 7a) show two clusters of sample IDs, suggesting that sites 2–4, 8, 11–14 share similar element concentration profiles, similarly for stations 1, 5–7, 9, and 10. Furthermore, there appear to be linear trends within each cluster, which could indicate a gradient or some form of ordered relationship among the element concentrations for those locations.

The first criterion chosen for Bayesian optimization was minimizing the *KL* divergence objective function between the high- and low-dimensional distributions. We obtained *Perp* = 3, the learning rate = 91.316, momentum = 0.677, and the best objective function = −0.037. Running the t-SNE with the new parameters, we found a different configuration of the data points represented by three clusters (Figure 7b).

**Figure 8:**
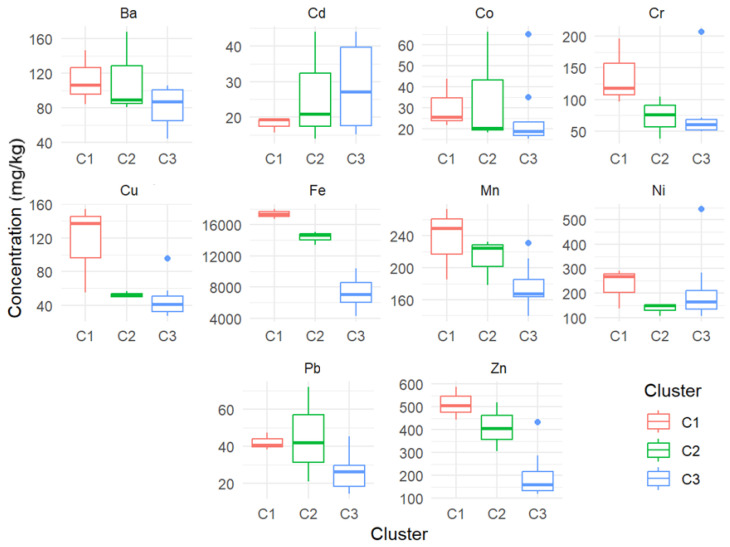
Concentrations of the elements in each cluster.

The quantitative analysis of the t-SNE results reveals important insights into clustering the sites based on the concentrations of various metals (Figure 8). Cluster 1 (containing sites 5, 6, and 10) and Cluster 2 (containing sites 1, 7, and 9) exhibit relatively small average intra-cluster distances of 14.32 and 15.25 units, respectively, indicating that the points within these clusters are closely packed and highly similar in terms of their element concentrations. In contrast, Cluster 3 (that contains sites 2–4, 8, 9, 11–14) shows a significantly larger intra-cluster distance of 44.02 units, suggesting more significant variability among the points within this cluster. The average inter-cluster distance of 101.33 units indicates that the clusters are well-separated, demonstrating clear distinctions between the different groups of samples. The compactness of Clusters 1 and 2 implies that the geographical locations represented by these clusters have similar compositions, likely due to similar environmental or geological conditions. The spread within Cluster 3 indicates that it encompasses a broader range of compositions, possibly from more diverse environments. The good separation between clusters validates the use of t-SNE and the defined clustering criteria, highlighting significant differences in dust compositions across the clusters.

The element concentration analysis reveals that different clusters have distinct element concentration profiles. For instance, C1 generally has higher concentrations for most elements, suggesting a unique dust composition compared to C2 and C3. C3, with the highest intra-cluster distance, shows the most variability in element concentrations, indicating a more diverse range of compositions within this cluster.

These patterns confirm that the t-SNE optimization has separated the data well and grouped samples with similar elemental profiles, enhancing the interpretability and reliability of the results.

The optimization of t-SNE parameters has provided better clustering results, as evidenced by the improved separation of clusters and the clear distinctions in element concentrations. The quantitative analysis supports the effectiveness of the optimization in terms of intra-cluster cohesion and inter-cluster separation. The element concentration analysis further validates these findings by showing distinct and meaningful differences in dust compositions across the clusters. Therefore, the optimization has successfully enhanced the quality and interpretability of the clustering results.

**Figure 9:**
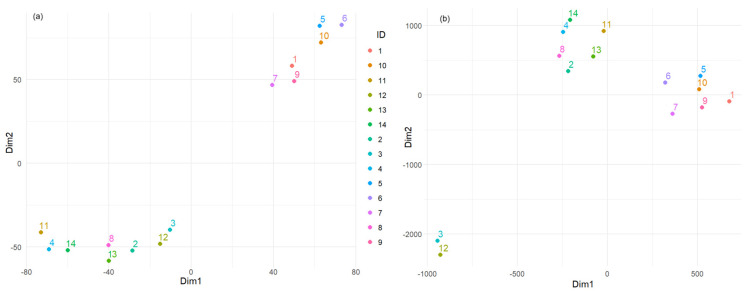
Results of t-SNE after optimization using (**a**) the silhouette score and (**b**) SME.

The second criterion for Bayesian optimization is maximizing the silhouette score [82]. This score assesses how well each point fits within its cluster. Scores close to one indicate that points are well-matched to their cluster and poorly matched to neighboring clusters. The results are presented in Figure 9a. Figure 7b and Figure 9a delimitate the clusters containing the sites (5, 6, 10) and (1, 7, 9) and show higher dissipations of the other elements contained in a third cluster.

The third criterion was to minimize the Sammon Mapping Error (SME) [83,84]. SME evaluates how well the distances in the low-dimensional space correspond to those in the high-dimensional space. Minimizing these errors ensures that the embedding accurately reflects the relationships between points. Figure 9b shows the results of this attempt. A clear delimitation of three groups appears: (3 and 12), (2, 4, 8, 11, 13, 14), and (1, 5, 6, 7, 9, 10). In this case, two clusters from the previous classifications—Figure 7b and Figure 9a—are put together (i.e., (1, 7, 9) and (5, 6, 10)), whereas the biggest one was split into two parts.

### 3.4. Results of Health Risk Assessment

The *ADD* by each path was computed initially (Table 5). The results indicate that the highest *ADD* corresponds to Fe and the lowest to Cd. The highest exposure to Fe and Cu is at D6, Ba, and Pb at D9, Cd and Co at D1, Ni at D8, Mn and Zn at D5, and Cr at D14. Exposure to Fe is much higher compared to other metals.

**Figure 10:**
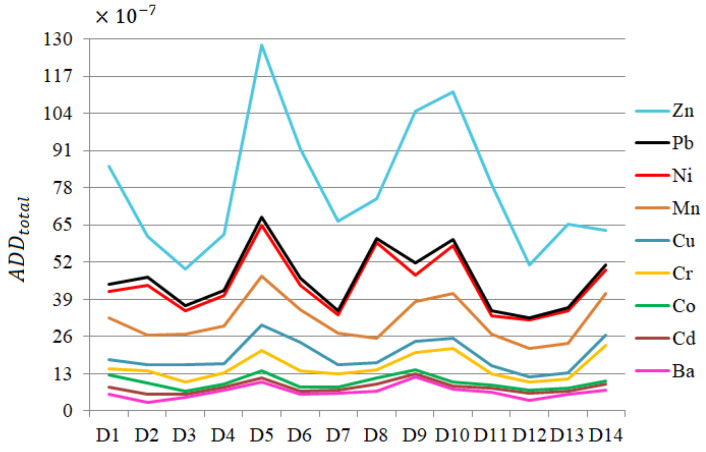
Chart of ${ADD}_{total}$ for all heavy metals but Fe.

**Figure 11:**
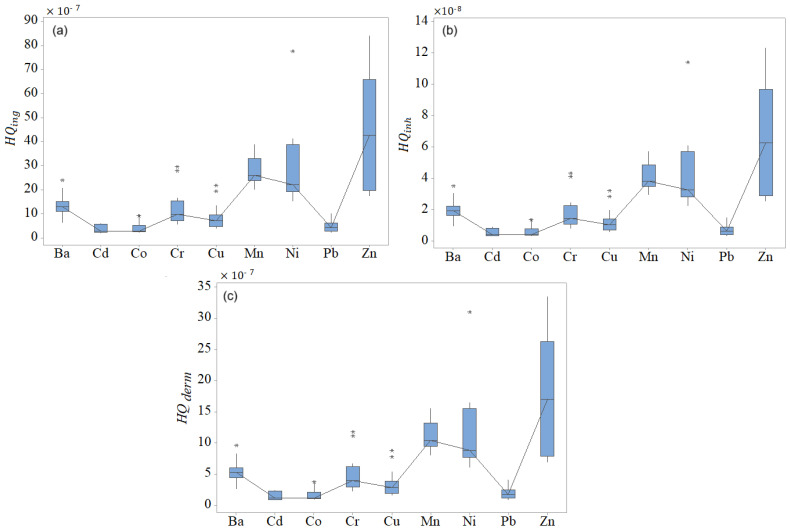
Boxplots of (**a**) ${HQ}_{ing}$, (**b**) ${HQ}_{inh}$, and (**c**) ${HQ}_{derm}$ for all heavy metals but Fe.

The chart of total *ADD* (${ADD}_{total}$) for all metals but Fe, obtained by summing up the *ADDs* by all paths, is contained in Figure 10. Among these elements, the highest ${ADD}_{total}$ corresponds to Zn, followed by Pb and Ni, with local peaks at D5 and D10. The ${ADD}_{total}$ for Fe is about 100 times higher compared to those of other heavy metals. The *HQs* for all metals but Fe are presented in Figure 11. ${HQ}_{ing}$ for Fe is between 6.10 $\times \;10^{-5}$ and 2.57 $\times \;10^{-4},$ the highest compared to those of other elements (Figure 11a).

For Fe, ${HQ}_{inh}$ belongs to the interval [8.97 × 10^−7^, 3.78 × 10^−6^], and ${HQ}_{derm}$ was between 24.33 × 10^−6^ and 102.584 × 10^−6^. Overall, the highest values of *HQs* correspond to Fe, Zn, Ni, and Mn. Outliers (represented by stars in Figure 11) are noticed for all *HQs* corresponding to Ba, Co, Cr, Cu, and Ni, indicating inhomogeneous distributions of *HQs*. Thus, the health risk related to exposure to these elements is highly variable at the spatial level.

**Figure 12:**
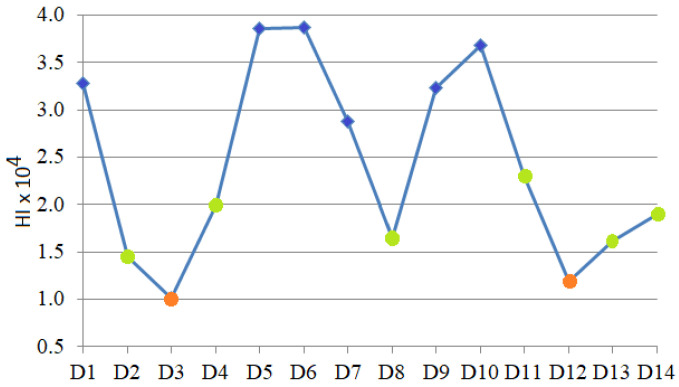
*HI* for the sampling sites.

Figure 12 contains the *HI* chart for the sampling sites. The points are colored differently according to the *HI* values. The lowest *HI* was obtained for 3 and 12, whereas the highest were obtained for 5, 6, and 10, followed by 1, 7, and 9 (in blue). The rest (in green) correspond to sites 2, 4, 8, 11, 13, and 14. This result is in concordance with the clustering obtained after using the SME. 

The lowest *HI* was found near green areas in zones 3 and 12. In contrast, the highest was noticed in the high-populated zones, close to the promenades (near the beach), where the pollution from high traffic is accentuated by atmospheric transport (aerosols carrying PM2.5), as explained in [85]. All *HI* values are less than one, indicating no non-carcinogenic risk.

This research complements [86], which presents the analysis results of the dust collected indoors in various locations in Dubai. However, instead of evaluating the contamination level with different metals in the dust using the quality indicators (e.g., *Igeo*, *EF*, *PI*, *PLI*, ${PI}_{Nemerow}$), the present work assessed the potential impact of pollution on population health. The high values of quality indicators computed with respect to Fe and Zn indicate that the pollution with these elements originates from industry, given that the UAE does not have significant resources for these metals. Moreover, the previous study [86] indicated high pollution with Cu, Pb, Zn, and Ni in heavy traffic and industry zones. This article found the last three elements to have the highest total *ADD*.

Compared to the output of [16], the *HIs* in the sampling zones in the Ajman and Sharjah industrial areas are many times higher than those in Dubai: 36.88 (for Cd), 8321.49 (for Cr), 1167.76 (for Cu), 1967.61 (for Ni), 11724 (for Pb), and 117.10 (for Zn), respectively.

More analysis of the pollution level from a different viewpoint is presented in another study that is currently under review.

## 4. Conclusions

The goal of this article was twofold. First, we aimed to evaluate the pollution intensity at 14 sampling points from Dubai based on the concentrations of 10 heavy metals. Secondly, we addressed the non-carcinogenic impact of pollution on the population’s health at the same locations.

To achieve the first goal, the dataset’s dimensionality reduction was performed by PCA, leading to the extraction of the most significant four PCs. The FA indicated that a two-factor model can adequately explain pollutants’ concentration variability. The t-SNE clustered the data series, whereas its optimization helped identify the similarities and differences between the dust content at the various sampling sites. It revealed that certain elements are prevalent in specific clusters, underlying environmental or geological factors influencing dust composition.

PCA performed for the dataset’s dimensionality reduction led to extracting the four most significant PCs.

*ADD* combined with the clustering results revealed the following:

- Extreme *ADDs*—the minimum for Cr and Cd, and maximum for Ba, Co, and Pb were computed for sites 1, 7, and 9 (belonging to the same cluster in Figure 7b);
- The *ADDs* for Fe and Pb reached their minimum at sites 3 and 12 (clustered together in Figure 9b);
- The maximum *ADD* for Fe and Pb were found at sites 5 and 6 (clustered together in Figure 7b);
- The *HI* values indicate a concordance between the clusters provided after t-SNE optimization and the magnitude of the non-carcinogenic risk to the population.

This information is valuable for interpreting the t-SNE optimization results and understanding the observed clusters’ real-world implications. It means that while clustering by itself would not be relevant, optimizing and using it together with the Hazard Index gives a correct image of the extent of pollution and its impact on population health. Moreover, it can be utilized as an early warning instrument for increased pollution, which can be used to take measures to maintain a clean environment.

Given the promising results of this approach, future studies will be developed to validate the findings using larger databases and testing other clustering optimization techniques and pollution indices. The study will emphasize the importance of statistical tools for better mapping the places where urgent measures are necessary for keeping safe living places for the population.

## Figures and Tables

**Table 1:** The exposure factors for adults.

Factor	Definition	Value
*c*	concentration of the heavy metal in the sample [mg/kg] computed here	
*R_ing_*	dust ingestion rate [mg/day]	100
*AT*	average time [day]	365 × ED
*BW*	mean weight of body [kg]	70
*EF*	frequency of exposure [days/year]	365
*ED*	duration of exposure [year]	24
*SA*	surface of the skin in contact with the dust [cm^2^]	5700
*R_inh_*	rate of inhalation [m^3^/day]	20
*SL*	factor of skin adherence for dust [mg/cm^2^]	0.07
*ABS*	factor of dermal absorption [-]	0.001
*PEF*	factor of particle emission [m^3^/kg]	1.36 × 10^9^

**Table 2:** *RfD* [mg/kg/day] used in this study for the analyzed metals.

Metal	Ingestion	Dermal	Inhalation
Ba	7 × 10^−2^	14 × 10^−3^	5 × 10^−4^
Cd	5 × 10^−4^	5 × 10^−6^	2 × 10^−5^
Co	3 × 10^−2^	5 × 10^−6^	6 × 10^−6^
Cr	3 × 10^−3^	15 × 10^−6^	1.4 × 10^−4^
Cu	4 × 10^−2^	12 × 10^−3^	1 × 10^−4^
Fe	0.7	2.2 × 10^−4^	7 × 10^−3^
Mn	2 × 10^−2^	8 × 10^−4^	5 × 10^−5^
Ni	2 × 10^−2^	54 × 10^−4^	2 × 10^−5^
Pb	14 × 10^−4^	42 × 10^−5^	1 × 10^−4^
Zn	0.300	0.0600	0.300

**Table 3:** *AIC* and *BIC* criteria as a function of the number of components.

Number of PCs	1	2	3	4
*AIC*	102.13	86.277	74.23	62.12
*BIC*	102.77	87.55	76.14	64.68

**Table 4:** FA analysis.

Metal	ML2	ML3	ML1	h2	u2	Com
Cd	−0.45	0.03	0.64	0.59	0. 413	1.8
Cr	−0.15	0.83	−0.15	0.62	0.383	1.1
Cu	0.53	0.04	−0.08	0.31	0.688	1.1
Ni	−0.11	−0.08	0.37	0.17	0.832	1.3
Pb	0.47	0.23	0.31	0.49	0.510	2.2
Co	0.09	−0.03	0.99	1.00	0.005	1.0
Ba	0.43	0.41	−0.21	0.58	0.415	2.5
Fe	0.86	0.11	0.07	0.86	0.137	1.0
Mn	0.26	0.77	0.18	0.91	0.093	1.3
Zn	0.97	−0.03	−0.02	0.91	0.091	1.0

**Table 5:** *ADD* × 10^8^ by various paths.

	${ADD}_{ing}\times 10^{8}$			${ADD}_{inh}\times 10^{8}$			${ADD}_{derm}\times 10^{8}$		
Metal	Min/Site	Max/Site	Mean	Min/Site	Max/Site	Mean	Min/Site	Max/Site	Mean
Ba	4.430	16.900	9.470	0.931	3.540	1.990	25.300	96.100	54.000
	D2	D9		D2	D9		D2	D9	
Cd	$9.98\times 10^{-3}$	$3.15\times 10^{-2}$	$1.85\times 10^{-2}$	0.294	0.926	0.544	7.970	25.100	14.800
	D7	D1, D12		D7	D1, D12		D7	D1, D12	
Co	0.663	2.85	1.230	8.820	37.900	16.4	0.325	1.400	0.605
	D12	D1		D12	D1		D12	D1	
Cr	0.164	0.889	0.383	0.803	4.360	1.880	0.803	118.000	50.900
	D1	D14		D1	D14		D1	D14	
Cu	1.540	8.810	3.590	0.576	3.240	1.320	15.400	87.900	35.800
	D12	D6		D12	D6		D12	D6	
Fe	427.00	1800	1090.00	243.000	1030.00	625.00	243.00	1030.00	623.000
	D3	D6		D3	D6		D3	D6	
Mn	3.980	7.820	5.640	2.930	5.750	4.150	79.500	156.000	113.000
	D8	D5		D8	D5		D8	D5	
Ni	3.020	15.500	5.760	2.220	11.400	4.230	60.300	310.000	115.000
	D11	D8		D11	D8		D11	D8	
Pb	$2.91\times 10^{-2}$	0.145	$6.76\times 10^{-2}$	8.300	41.200	19.300	0.306	1.529	0.710
	D12	D9		D12	D9		D12	D9	
Zn	518.00	2500.00	1340.00	2.540	12.400	5.590	0.689	33.500	179.000
	D14	D5		D14	D5		D14	D5	

## Data Availability

Data will be available on request from the second author.
